# Generation of Marker- and/or Backbone-Free Transgenic Wheat Plants via *Agrobacterium*-Mediated Transformation

**DOI:** 10.3389/fpls.2016.01324

**Published:** 2016-09-21

**Authors:** Gen-Ping Wang, Xiu-Dao Yu, Yong-Wei Sun, Huw D. Jones, Lan-Qin Xia

**Affiliations:** ^1^Department of Plant Gene Resources and Molecular Design, Institute of Crop Sciences, Chinese Academy of Agricultural Sciences (CAAS)Beijing, China; ^2^Cereal Crops Research Laboratory of Hebei Province, National Millet Improvement Center, Institute of Millet Crops, Hebei Academy of Agriculture and Forestry SciencesShijiazhuang, China; ^3^Translational Genomics for Plant Breeding, Institute of Biological, Environmental and Rural Sciences, Aberystwyth UniversityAberystwyth, UK

**Keywords:** *agrobacterium*-mediated transformation, dual binary vector, durum wheat (*triticum turgidum* L. var. *durum* cv stewart 63), genetically modified (Gm) wheat, marker- and backbone-free transgenic line

## Abstract

Horizontal transfer of antibiotic resistance genes to animals and vertical transfer of herbicide resistance genes to the weedy relatives are perceived as major biosafety concerns in genetically modified (GM) crops. In this study, five novel vectors which used *gusA* and *bar* as a reporter gene and a selection marker gene, respectively, were constructed based on the pCLEAN dual binary vector system. Among these vectors, 1G7B and 5G7B carried two T-DNAs located on two respective plasmids with 5G7B possessing an additional *virGwt* gene. 5LBTG154 and 5TGTB154 carried two T-DNAs in the target plasmid with either one or double right borders, and 5BTG154 carried the selectable marker gene on the backbone outside of the T-DNA left border in the target plasmid. In addition, 5BTG154, 5LBTG154, and 5TGTB154 used pAL154 as a helper plasmid which contains Komari fragment to facilitate transformation. These five dual binary vector combinations were transformed into *Agrobacterium* strain AGL1 and used to transform durum wheat cv Stewart 63. Evaluation of the co-transformation efficiencies, the frequencies of marker-free transgenic plants, and integration of backbone sequences in the obtained transgenic lines indicated that two vectors (5G7B and 5TGTB154) were more efficient in generating marker-free transgenic wheat plants with no or minimal integration of backbone sequences in the wheat genome. The vector series developed in this study for generation of marker- and/or backbone-free transgenic wheat plants *via Agrobacterium*-mediated transformation will be useful to facilitate the creation of “clean” GM wheat containing only the foreign genes of agronomic importance.

## Introduction

Wheat (*Triticum aestivum* L.) is one of the major staple food crops grown worldwide on more than 17% of the cultivatable land and has made a significant contribution to the increase in global food supply (Xia et al., 2012). However, global wheat production is under pressure from changing climates and the increasing financial and environmental costs associated with chemical inputs. Present attempts to raise the yield and qualities of wheat are being confronted by slow progress in the genetic improvement of yield potential and stability through conventional breeding. Outbreaks of drought, fungal, and insect pests causing substantial losses are thus being reported regularly (Xia et al., 2012). Therefore, there is an urgent need to mitigate the effects of the stresses on wheat yield through the use of tolerant or resistant varieties which could be financially and environmentally beneficial. Genetically modified (GM) wheat opens up new opportunities to meet these challenges (Xia et al., 2012).

*Agrobacterium*-mediated transformation and microparticle bombardment are two widely used methods for wheat genetic transformation (Jones, 2005; Bhalla, 2006). Like other plants, *Agrobacterium*-mediated genetic transformation of wheat also requires the inclusion of selectable marker genes (SMGs) that enable transformed plants or cells to be identified. In most cases, antibiotic or herbicide resistance marker genes are preferred because they tend to be more efficient (Malnoy et al., 2010), although others, based on availability of required nutrient or a color marker are also used (Joersbo and Okkels, 1996; Okkels et al., 1997; Haldrup et al., 1998; Lucca et al., 2001; Stewart, 2001; Wright et al., 2001). Once the transgenic plants have been generated, the SMGs become superfluous and undesirable, and SMGs that remain in commercialized events have raised concerns regarding their potential transfer to other organisms and their safety. For example, horizontal transfer of antibiotic resistance genes to animal and human gut bacteria, and vertical transfer of herbicide resistance genes to the weedy relatives are perceived as major biosafety concerns in genetically engineered crops (Dale et al., 2002; Rao et al., 2011). Furthermore, developing elite lines and varieties usually requires incorporating multiple desirable traits and foreign genes into a variety, a process called gene pyramiding or stacking. If this process is attempted by retransformation, the presence of a SMG prevents the use of the same marker system for any successive round of transformation (Kapusi et al., 2013). Moreover, in an ideal model, the T-strand to be transferred from *Agrobacterium* to plant cell is synthesized from DNA delimited by the left and right T-DNA border repeats (LB and RB), and then integrated into the plant genome intact without any vector backbone sequences. However, it has been known for some time that the partial T-DNAs or DNA sequences from the binary plasmid external to the T-DNA borders can also be incorporated into the plant genome due to the possible imprecise nicking function of VirD2 nuclease which causes the premature T-strand termination or read-through into vector backbone (Ooms et al., 1982; Martineau et al., 1994; Cluster et al., 1996; Van der Graaff et al., 1996; De Buck et al., 2000; Yin and Wang, 2000; Sallaud et al., 2003; Wu et al., 2006). Therefore, to develop GM wheat for future commercialization, the ideal situation is to generate elite commercial lines of GM wheat without SMGs and bacterial backbone DNA sequences.

So far, several strategies have been elaborated to produce marker-free transgenic plants. One strategy is to use a site-specific recombinase-mediated marker excision system, such as Cre/lox (Hoess et al., 1982; Hoess and Abremski, 1985), FLP/FRT (Cox, 1983; Senecoff et al., 1985), and R/Rs (Araki et al., 1985), to eliminate SMGs and avoid pollen-mediated transmission of transgenes and facilitate subsequent gene stacking by re-transformation (Ow, 2001; Mlynárová et al., 2006; Verweire et al., 2007; Moon et al., 2011). Simultaneous application of the minimal gene cassette and cold-inducible Cre/lox recombination system in wheat generated marker-free transgenic wheat plants through bombardment (Mészáros et al., 2015), although little molecular evidence on the integration and genetic stability of transgene was available in that study. A second strategy is to use transposon-mediated transgene repositioning through intra-chromosomal homologous recombination to remove SMGs via segregation in the progenies of GM plants (Goldsbrough et al., 1993; Ebinuma et al., 1997; Zubko et al., 2000; Cotsaftis et al., 2002). However, the frequency of homologous recombination in flowering plants or transposon activity in heterologous hosts is very low (Yau and Stewart, 2013; Nishizawa–Yokoi et al., 2014), and thus limits the future application of this technology in agriculturally important crop plants. A third strategy involves the introduction of the SMGs and the gene of interest (GOI) separately either on different T-DNAs or on the backbone of the vector. Provided that the two transgenes are not linked in a *cis* configuration, the SMG insertion segregates independently of the GOI insertion, allowing for the recovery of marker-free plants in the subsequent generation (McKnight et al., 1987). In these vector systems, two T-DNAs can either be presented in one-vector/one-strain (Komari et al., 1996; Xing et al., 2000), two-vectors/one-strain (Framond et al., 1986; Daley et al., 1998), or two vectors/two strains (McKnight et al., 1987; De Block and Debrouwer, 1991; De Neve et al., 1997). In addition, a single binary vector containing transgenes in both the T-DNA and the backbone (RB-GOI-LB-SMG) had been used for maize (*Zea mays*) transformation; the maker-free transgenic progeny was recovered by co-transformation of the T-DNA and backbone components following segregation (Huang et al., 2004). Moreover, a double right-border (DRB) binary vector carrying two copies of T-DNA RB sequences flanking a SMG, followed by a GOI and one copy of the left border sequence (RB-SMG-RB-GOI-LB) have also been used in rice and tobacco transformation to generate marker-free transgenic lines (Lu et al., 2001; Matheka et al., 2013). Unlinked integration of T-DNA strands generated from each RB and terminated at the same LB in rice cells ensured the efficient segregation of the T-DNA containing both the rice ragged stunt virus (RSSV) gene and the selectable hygromycin resistance gene in the progeny of transgenic plants (Lu et al., 2001). In summary, the objectives of these strategies are to obtain marker-free plants in the subsequent generation of transgenic plants in which the two T-DNAs have integrated at unlinked loci in the plant genome, such that the SMG insertion segregates independently of the GOI.

In addition, a series of vectors with improved transformation characteristics have been developed and used in plant transformation so far. For example, vectors have been engineered to improve stable or transient transformation efficiency with the addition of virulence (*vir*) genes such as *virGwt* or a Komari fragment which contains *virB, virC*, and *virG*^542^ simultaneously (Komari, 1990; Hiei et al., 1994; Vain et al., 2004), suppressors of gene silencing (Hellens et al., 2005) or overdrive sequences near the border sequences (Podevin et al., 2006). Binary vectors with small T-DNAs (Düring, 1994; Barrell and Conner, 2006), tandem border repeats (Kuraya et al., 2004; Podevin et al., 2006), or containing only plant-derived DNA (Rommens, 2004) have also been engineered to minimize the insertion of unwanted DNA sequences into the plant genome. To facilitate the elimination of the redundant sequences and the SMGs, a new multifunctional dual binary vector system, pCLEAN, for *Agrobacterium*-mediated plant transformation has been developed by Thole et al. (2007). The pCLEAN vectors are developed based on the widely used pGreen/pSoup system (Hellens et al., 2000), and have been designed to enable the delivery of multiple transgenes from distinct T-DNAs and/or vector backbone sequences while minimizing the insertion of superfluous DNA sequences into the plant nuclear genome as well as facilitating the production of marker-free plants in tobacco and rice (Thole et al., 2007).

Generation of elite GM wheat lines without SMGs, deleterious backbone sequences and/or bacterial DNA sequences would be highly desirable to develop GM wheat for future commercialization to meet diverse challenges in wheat production. However, although the attempts to establish a marker- and/or backbone-free *Agrobacterium*-mediated transformation system have been documented in other crop plants, it has never been reported in wheat so far. In this study, by using *gusA* as a reporter gene and *bar* as a SMG, a series of modified vectors were constructed based on the pCLEAN vector system. Among them, five dual binary vectors were used in wheat *Agrobacterium*-mediated transformation. The aim of this study is to evaluate the differences of these five dual binary vectors in the following aspects such as the final transformation efficiencies, the frequencies of generation of marker-free transgenic wheat progeny following segregation, and the integration of backbone sequences beyond the transgene in the transgenic lines, in order to provide an insight into generation of marker- and/or backbone-free clean GM wheat via *Agrobacterium*-mediated transformation.

## Materials and methods

### Construction of binary transformation vectors

The basic binary vectors used in this study were pCLEAN-G181, pCLEAN-G185, and pCLEAN-S167 plasmids which were kindly provided by Dr. Philippe Vain, John Innes Centre, UK, and constructed using standard DNA cloning methods in the *Escherichia coli* strains DH5a (Sambrook et al., 1989). In general, the basic Gateway compatible vectors of pCG181-1, pCG185-1, and pCS167-1 were constructed using the reading frame A (rfA) of the Gateway vector conversion system (Invitrogen, USA). In each case, pCG181-1 and pCG185-1 were constructed by inserting the rfA into the *Not*I-digested, blunted and dephosphorylated pCLEAN-G181 and pCLEAN-G185 vectors, respectively; pCS167-1 were constructed by inserting the rfA into the *Pac*I-digested, blunted and dephosphorylated pCLEAN-S167 plasmids. The pCG185-1 vector was further used to develop pCG185-2, pCG185-3, and pCG185-4 plasmid by inserting *bar* cassette with various borders of T-DNA into the *Pme*I site of backbones (Figure S1B). Firstly, *bar* cassette (Ubi+intron:*bar*:nos) was amplified (Fu*bar*_(CACC)_/Rt*bar*) from pGreenII-UB plasmid (Hellens et al., 2000) with high fidelity PrimerStar polymerase (TakaRa, Japan) and Fubar_(CACC)_/Rtbar primer set. The resulting PCR products were captured by topoisomerase-mediated cloning into the pENTR/D-TOPO vector (Invitrogen, USA), to produce pENTR-*bar*, and then transferred into pCG185-1 plasmid through LR recombination reaction, creating intermediate plasmid pCG185-1*bar*. pENTR-*bar* was linearized by *Ssp*I digestion prior to LR reaction, since both entry clone and destination vector code for kanamycin resistance. On the other hand, the resulting PCR products were cloned into the *Pme*I site of pCG185-1 plasmid outside the LB of T-DNA, creating pCG185-2. Secondly, two essential segments of LB-*bar* cassette (LB-Ubi+intron:*bar*:nos) and 2LB-*bar* cassette-RO (2LB-Ubi+intron:*bar*:nos-RB-Overdriver) fragment amplified from pCG185-1*bar* were inserted into the *Pme*I site of pCG185-1 plasmid, respectively, to generate pCG185-3 and pCG185-4 vectors (Figure S1C).

To introduce the *gusA* cassette into the above plasmid, the pENTR-*gusA* vector was constructed through topoisomerase-mediated cloning with the *gusA* cassette (Ubi+intron:*gusA*:nos) amplified from pAL156 plasmid with the Fu*gusA*/Rt*bar* primer set (He et al., 2010), then transferred into pCG181-1, pCG185-1, pCG185-2, pCG185-3, and pCG185-4 through LR recombination reaction, to generate the vectors pCG181-1G, pCG185-1G, pCG185-2G, pCG185-3G, and pCG185-4G (Figure S2). Plasmid pENTR-*gusA* was linearized by *Apa*I digestion prior to LR reaction and pENTR-*bar* and pCS167-1 were combined by LR reaction to produce pCS167-1B (Figure S2). The primer sets used in this section were shown in Table S2. All recombinant plasmids identified from individual colonies were verified by sequencing.

**Figure 1:**
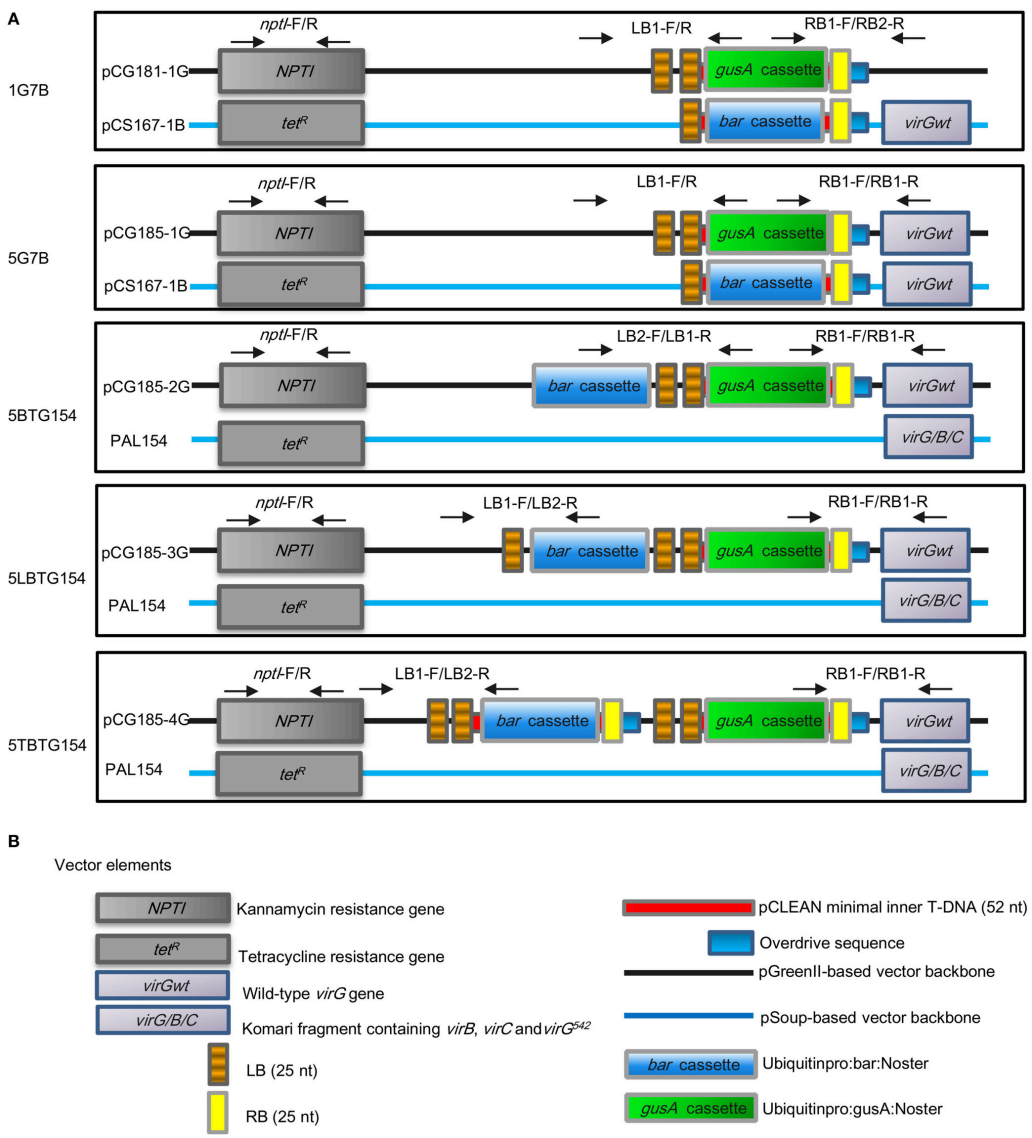
**Five sets of T-DNA vectors developed and tested in this study**. **(A)** The constructs of five different dual binary combinations; **(B)** Vector elements. The vector elements were cited from Thole et al. (2007) with minor modifications. Five different dual binary vectors were introduced into *Agrobacterium* strain AGL1, respectively. pCS167-1B and pAL154 have the pSa-Rep ori to provide replication functions *in trans* for the pCG181-1G, pCG185-1G, pCG185-2G, pCG185-3G, pCG185-4G vector series, respectively. In addition, pCS167-1B has a T-DNA harboring a *bar* expression cassette. Arrows show the location of the primer sets for detection of the integration of backbone sequences in T_0_ plants. The presence of *NPTI*, LB, and RB in the T_0_ plants derived from each vectors were detected by the indicated three primer sets.

Finally, five combinations of plasmids: pCG181-1G and pCS167-1B (1G7B), pCG185-1G and pCS167-1B (5G7B), pCG185-2G and pAL154 (5BTG154), pCG185-3G and pAL154 (5LBTG154), pCG185-4G and pAL154 (5TGTB154) were introduced into the *Agrobacterium tumefaciens* strain AGL1 (Lazo et al., 1991) by electroporation method (BIO-RAD Gene Pulse Xcell, Germany), respectively (Figure 1).

### Wheat transformation

Durum wheat (*Triticum turgidum* L. var. *durum* cv. Stewart 63) were grown in the field at the CAAS experimental station under normal field management conditions. Ears were harvested at 10–15 days after pollination for isolation of immature embryos (IEs). Immature seeds were sterilized first with 70% ethanol for 1 min, followed by 10% (v/v) Domestos (Lever Faberge, UK) for 15 min, and then rinsed three times with sterilized distilled water. IEs were isolated under aseptic conditions after removal of the entire axis. The length of IEs ranged from 0.8 to 1.5 mm. *Agrobacterium*-mediated transformation was performed following the protocol described by He et al. (2010). For each vector combination, the total numbers of immature embryos inoculated ranged from 1067 to 2000 with three replications (Table 1). Plants showing resistance to PPT were subjected to a PCR analysis and/or a GUS activity assay. Positive plants were transferred to soil and grown in a greenhouse under normal management.

**Table 1:** **Summary of the transformation experiments using different binary vectors**.

	**1G7B**	**5G7B**	**5BTG154**	**5LBTG154**	**5TBTG154**
a	1067	1714	2000	1846	1344
b	16	48	14	24	54
c	1.5 ± 0.3% A	2.8 ± 0.8% A	0.7 ± 0.2% B	1.3 ± 0.2% A	4.0 ± 2.1% A
d	37.5% (6/16)	56.3% (27/48)	92.9% (13/14)	87.5% (21/24)	63.0% (34/54)
e	100% (6/6)	88.9% (24/27)	100% (13/13)	95.2% (20/21)	88.2% (30/34)
f	16.6% (1/6)	29.2% (7/24)	38.4% (5/13)	45.0% (9/20)	30.0% (9/30)
g	13.3% (2/15)	23.4% (44/188)	21.4% (24/112)	25.4% (47/185)	19.7% (38/193)

*a, No. of inoculated embryos; b, No. of independent T0 lines generated; c, Transformation efficiency (Independent events generated/embryos inoculated; standard ± deviation); d, The frequency of co-transformed events (number of co-transformants/independent lines generated); e, The frequency of co-expression (Events with both GOI and SMG co-expressed/co-transformed events); f, The frequency of GOI and SMG segregation in T1 progenies (Events in which GOI and SMG segregated in T1 progenies/events with co-expression of both GOI and SMG in T0 generation); g, The frequency of SMG free progenies (the number of SMG free plants/total number of analyzed T1 plants)*.

*The transformation efficiency values marked by different letters are statistically significant at P < 0.05*.

### PCR analysis

Genomic DNA was extracted from wheat leaf with the DNAquick Plant System Kit (Tiangen, Beijing, China). PCR was used to confirm the presence of transgenes in the primary transformants and their progeny. The presence of the *bar* gene was determined by amplification of a 421 bp fragment using the primer sets *bar*-F/*bar*-R (Table S2). The presence of *gusA* gene was determined by amplification of a 1051 bp fragment using the primer sets *gusA*-F/*gusA*-R (Table S2). PCR analysis was carried out in a reaction volume of 25 μl and the annealing temperature was 57°C for *bar* and 62°C for *gusA*, respectively. At least two replicates were carried out for each sample.

### PCR analysis for backbone sequence detection

Primer sets were designed to check the vector backbone sequence of independent T_0_ plants. Four primer pairs were used to detect 771, 1268, 511, and 561 bp fragments outside *gusA* T-DNA at the left border of 1G7B, 5G7B, 5BTG154, 5LBTG154, and 5TBTG154, respectively; one primer pair was used to detect the *NPTI* gene and another two primer sets were used to detect sequences beyond the T-DNA right border of 1G7B, 5G7B, 5BTG154, 5LBTG154, and 5TBTG154, respectively. Sequence of primer pairs, annealing temperature and expected fragment sizes are shown in Table S3. At least two replicates were carried out for each PCR analysis.

### Assay for GUS activity

Transient GUS expression was determined using the histochemical GUS assay as described by Wu et al. (2003). Explants were incubated overnight at 37°C in X-Gluc buffer containing 1 mM X-Gluc, 100 mM sodium phosphate buffer (pH 7.0), 0.5 mM potassium ferricyanide, 0.5 mM potassium ferrocyanide, and 0.1% (v/v) Triton X-100. Blue foci were counted with the aid of a microscope. T-DNA delivery was assessed by counting embryos that had at least one GUS focus and then counting the number of foci per embryo. To assay for stable expression, calli, shoots, leaf fragments, and seeds from regenerating plants were also incubated in X-Gluc buffer overnight at 37°C, and then chlorophyll was removed from tissues using 70% ethanol under light for 12–24 h.

### Ammonium assay for *bar* gene expression

The ammonium assay, for qualitative detection of phosphinothricin acetyltransferase (PAT) activity was performed according to Wu et al. (2006). Leaf pieces (3 × 6 mm) from transgenic and control plants at three-five leaves stage, were immersed together into 1 ml incubation solution at 23–25°C for 6 h under light. For each sample, a 200 μl incubation solution was transferred into a single well of a 24-well plate and mixed with 1 ml of the two reagent mixes followed by incubation at 37°C for 20 min in the dark. Finally, the color of the solution was judged qualitatively as either positive or negative. Leaf samples without *bar* gene expression gave jewelry blue to dark blue color, whereas those expressing the *bar* gene gave an obviously lighter color of pale yellow.

### Southern blot analysis

Genomic DNA was extracted from young leaf tissue of T_0_ plants using CTAB method as described by Sambrook et al. (1989). About 35 μg of genomic DNA was digested overnight with the restriction enzyme, fractionated on 0.8% agarose gel in 1 × TAE buffer for 12–16 h at 60 V, and then blotted to Hybond-N+ membranes (Amersham, UK). Prehybridization, hybridization, washing, and detection of the membranes were conducted using the digoxigenin (DIG) High Prime DNA Labeling and Detection Starter Kit I (Roche, Germany). DNA probes for the *gusA* gene and *bar* gene were synthesized using the primer sets *gusA*F/*gusA*R and *bar*F/*bar*R (Table S3) with a PCR DIG probe synthesis kit (Roche, Germany).

### Segregation analysis

T_0_ plants with ~30 seeds or more were harvested for segregation analysis. At the 3-/4-leaf stage, leaf material was harvested and subjected to bar gene ammonium assay (Wu et al., 2006) and GUS assay if necessary. Seeds with no histochemical GUS staining were confirmed to be null segregants by PCR.

### Statistical analysis

Statistical analysis was performed using SPSS software. Data sets were compared by using Student's *t*-test. Significance (*P*-value) was evaluated at the 5% level for all comparisons. For each treatment, the standard error of the mean (SE) was calculated based on at least three biological replicates.

## Results

### Construction of the modified pCLEAN binary vectors for wheat *Agrobacterium*-mediated transformation

The pCLEAN vectors were developed from the dual binary system pGreen/pSoup which enables the coexistence of pCLEAN-G and pCLEAN-S binary vectors within a single *Agrobacterium* strain (Thole et al., 2007). To facilitate cloning of GOI or SMG into the pCLEAN vectors to generate marker-free transgenic plants, three pCLEAN gateway-compatible vectors pCG181-1, pCG185-1, and pCS167-1 (GenBank Accession No. KT163016, KT163017, and KT163018, respectively), with the T-DNA region harboring gateway recombination sites (attR1 and attR2) for introducing GOI and SMG expression cassettes, were constructed in this study based on pCLEAN-G181, pCLEAN-G185, and pCLEAN-S167 vectors (GenBank Accession No: EU186083, EU186084, and EU186086, respectively; Figure S1A). Furthermore, three vectors with selection marker *bar* gene cassette located in the backbone either without a LB, with one LB, or within a T-DNA with two LBs, were constructed based on the pCLEAN-G185 vector. These vectors were referred as pCG185-2, pCG185-3, and pCG185-4, respectively, and were designed to exploit vector backbone transfer and separate T-DNA transfer in one plasmid to produce marker-free transgenic plants (Figure S1B). For example, pCG185-2 was developed to exploit the fact that *Agrobacterium* frequently delivers a range of DNA fragments (i.e., T-DNA alone or T-DNA along with vector backbone sequence) at linked or unlinked loci into the plant nuclear genome. This enables the SMGs located outside the T-DNA (in the vector backbone) to be co-transformed with GOIs located inside the T-DNA. When T-DNA and T-DNA plus backbone fragments integrate at different loci, progeny plants free of SMG (i.e., containing only the GOI) can be recovered. Furthermore, the pCG185-3 vector was developed in which a SMG was flanked between two T-DNA LBs, following by an integrative T-DNA harboring GOI cassette. Two types of T-DNA inserts, one initiated from the RB containing both the GOI and SMG, and the other from the RB containing only the GOI, were expected to be produced and integrated into the genome at linked or unlinked loci in the plant's genome. Finally, the pCG185-4 vector harbors two separate T-DNAs, enabling the SMG and the GOI to be located within two separate T-DNAs with one RB and two LBs in a single binary vector. In this case, two independent T-DNAs can be produced and integrated into plant genome at linked or unlinked loci. These three vectors were tested for their ability to produce marker-free plants when paired with the pSoup vector pAL154 to facilitate T-DNA transfer.

To evaluate the efficiency of this modified pCLEAN vector system on generation of marker-free transgenic lines in wheat after *Agrobacterium*-mediated transformation, a *gusA* expression cassette was introduced by using a gateway system into the T-DNA of the vector series developed in this study to generate plasmids pCG181-1G, pCG185-1G, pCG185-2G, pCG185-3G, and pCG185-4G. The *bar* gene expression cassette was introduced into the helper plasmid pCS167-1 through a gateway system to generate pCS167-1B (Figure S2). The various pCLEAN and pSoup vector combinations used in wheat *Agrobacterium*-mediated transformation in this study were given the following identifiers; 1G7B (combination of pCG181-1G and pCS167-1B), 5G7B (pCG185-1G and pCS167-1B), 5BTG154 (pCG185-2G and pAL154), 5LBTG154 (pCG185-3G and pAL154), and 5TBTG154 (pCG185-4G and pAL154) (Figure 1). As indicated in Figure 1, in addition to the advantages of the pCLEAN vector system as described by Thole et al. (2007), these five vectors were engineered to improve plant transformation efficiency and generate clean transgenic plants. For example, the 1G7B and 5G7B combinations each contain two plasmids in which each harboring an independent T-DNA with *gusA* or *bar* gene cassettes, respectively. Compared with 1G7B, 5G7B had an additional *virGwt* and located on backbone of pCG185-1G. The 5BTG154 and 5LBTG154 combinations had the structure of “RB-*gusA*-2LB-*bar*” in pCG185-2G plasmid and “RB-*gusA*-2LB-*bar*-LB” in pCG185-3G plasmid, respectively, and were both matched with a helper plasmid pAL154 which contains a 15.2 Kb Komari fragment harboring extra *virB, virC* and *virG542* (*virB/C/G*) from Ti plasmid pTiBo542, which has been demonstrated to have a role in enhancing T-DNA delivery (Komari, 1990; Vain et al., 2004). The 5TBTG combination contained two independent T-DNAs, each with either *gusA* or *bar* expression cassette in pCG185-4G plasmid, and matched with pAL154 (Figure 1). In these combinations, pCG185-1G, pCG185-2G, pCG185-3G, pCG185-4G, and pCS167-1B all contained one *virGwt* gene and a Komari fragment. Thus, three versions of *vir* gene(s) in these five vectors were incorporated, these were 1G7B with one *virGwt*, 5G7B with two *virGwt*, 5BTG154, 5LBTGTB154, and 5TBTG154 with both *virGwt* and *virB/C/G* (Figure 1). These vectors were transformed into *Agrobacterium* strain AGL1, which contains the pTiBo542 as a resident Ti plasmid, and then used for wheat transformation (Wu et al., 2003).

### Generation of transgenic wheat lines using different dual binary vectors

At first, the effect of *vir* gene(s) on GUS transient expression and T-DNA delivery efficiency was investigated, both in terms of the embryos that displayed at least one locus of GUS staining on the scutellum surface and also of the average number of GUS loci per explant as described by He et al. (2010). Higher *gusA* transient expression frequency was observed in the combinations of 5BTG154, 5LBTG154, and 5TBTG154 that contained *virGwt* in pCLEAN-G based vectors and *virB/C/G* in helper plasmid pAL154 (Figure S3). Additional *virGwt* on pCG185-2G in 5G7B increased T-DNA delivery efficiency with the mean number of blue spots per scutellum significantly higher than 1G7B (Figure S3B). These results indicated that the additional *vir* gene(s) indeed enhanced the efficiency of T-DNA delivery to immature embryos of wheat as observed by other reports (Wu et al., 2003; Vain et al., 2004).

Then, the independent transgenic wheat lines were generated through a series of transformation experiments by using immature wheat embryos under two rounds of glufosinate selection. The process of *Agrobacterium*-mediated durum wheat transformation was as shown in Figure S4. Independent wheat transgenic lines were generated from each vector combination. Numbers of T_0_ plants obtained varied with different vector combinations. The final transformation efficiencies of five dual binary vectors were as indicated in Table 1. The numbers of transgenic lines generated were 16, 48, 14, 24, and 54 for vectors 1G7B, 5G7B, 5BTG154, 5LBTG154, and 5TBTG154, with final transformation efficiencies of 1.5 ± 0.3, 2.8 ± 0.8, 0.7 ± 0.2, 1.3 ± 0.2, and 4.0 ± 2.1%, respectively. Compared to 5BTG154, a significant increase of the final transformation efficiency was observed in 5G7B, 5LBTG154, and 5TBTG154 (one tailed student's *t*-test, *P* < 0.05). In addition, the transformation efficiency of the combination 5G7B (2.8 ± 0.8%) was higher than that 1G7B (1.5 ± 0.3%). Furthermore, PCR analysis of these independent lines showed that the co-transformation efficiency of T_0_ plants that were positive for both the *gusA* and *bar* for 1G7B, 5G7B, 5BTG154, 5LBTG154, and 5TBTG154 combinations were 37.5, 56.3, 92.9, 87.5, and 63.0% (Table 1), respectively, suggesting either the separate integration of *gusA* and *bar*, or that selection escapes resulted from these vector combinations. The co-transformed lines which were positive for both the *gusA* and *bar* genes for 5BTG154 (92.9%) and 5LBTG154 (87.5%) combinations in which the *gusA* inside the T-DNA and *bar* located in the backbone indicated a different T-DNA delivery type. One possible explanation for the results of 5BTG154 might be that two types of insertion such as RB-*gusA*-LB-*bar* or RB-*gusA*-LB could be generated and integrated in the genome as suggested by Huang et al. (2004). For 5LBTG154, the structure of RB-*gusA*-LB-*bar*-LB could generate either RB-*gusA*-LB or RB-*gusA*-LB-*bar*-LB structure and integrated in the genome. Further expression analysis indicated that among the transgenic lines with both *gusA* and *bar*, 100, 88.9, 100, 95.2, and 88.2% lines derived from 1G7B, 5G7B, 5BTG154, 5LBTG154, and 5TBTG154 vectors, respectively, produced functional GUS and PAT proteins (Table 1).

**Figure 2:**
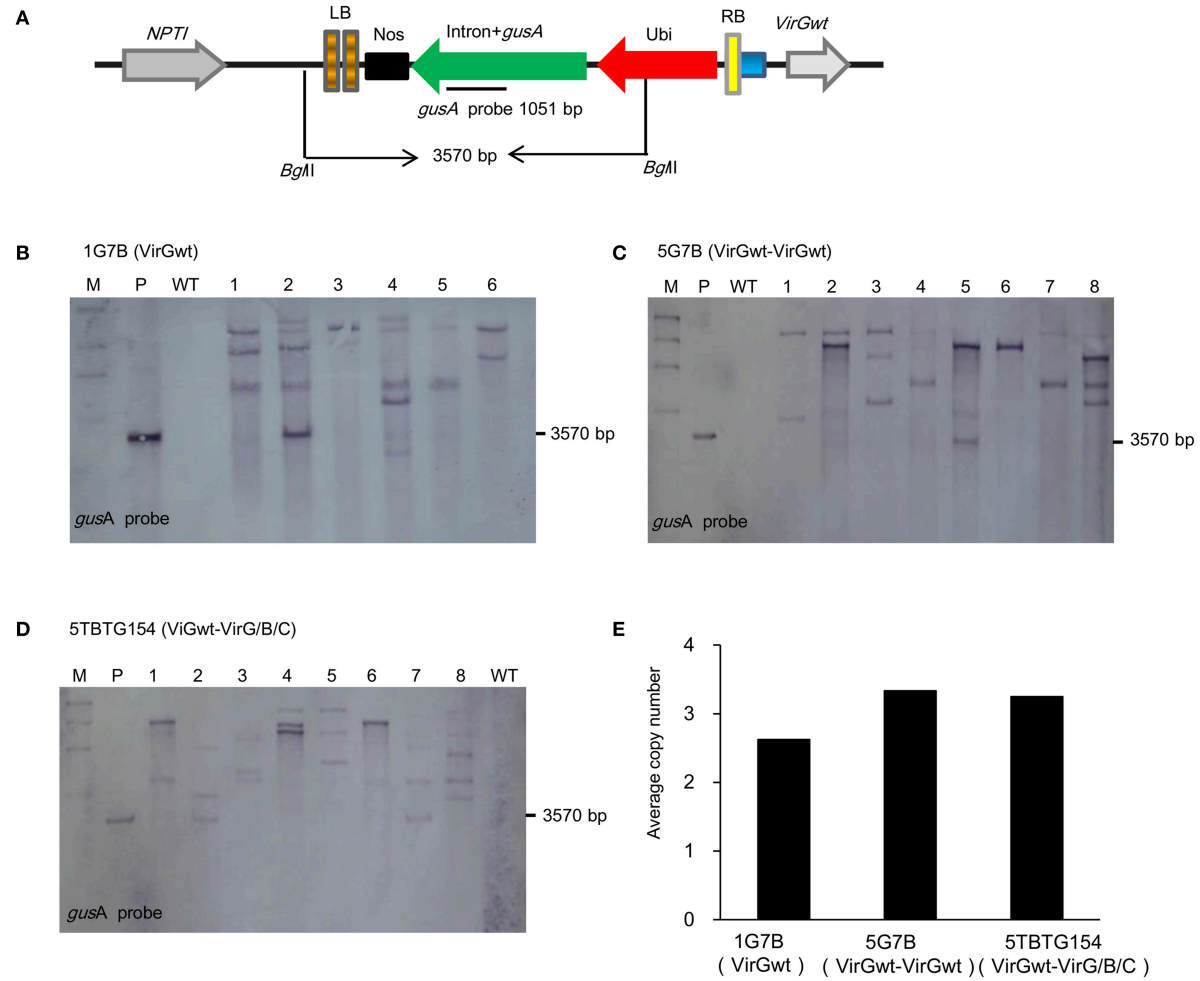
**Southern blot analyses of T_0_ wheat plants. (A)** Schematic show of the *gusA* gene expression cassettes, *Bgl*II restriction sites and T-DNA borders. **(B)** 1G7B vector. **(C)** 5G7B vector. **(D)** 5TBTG154 vector. **(E)** Effect of different additional *vir* genes combinations on transgene structure in T_0_ wheat plants which derived from 1G7B, 5G7B, or 5TBTG154 containing the same *gusA* cassette in same T-DNA but with different *vir* gene(s). Plant genomic DNA was digested using restriction enzyme *Bgl*II which cuts in the middle of the T-DNA in the *gusA* expression unit. Membranes were probed with the *gusA* gene. Average copy number was estimated using the number of hybridizing fragments.

Furthermore, in order to analyze the effect of additional *vir* gene(s) on the integration patterns and copy number of the *gusA*, co-integrated lines from 1G7B, 5G7B, and 5TBTG154 were tested in randomly selected lines by Southern blot analysis since the combinations of 5BTG154 and 5LBTG154 had the same *vir* gene(s) version as that of the 5TBTG154. Both plasmid and genomic DNA were digested by *Bgl*II and hybridized with the 1051 bp *gusA* probe. *Bgl*II cuts twice in the pCG185-1G plasmid and releases a 3570 bp fragment containing the *gusA*. As expected, *Bgl*II digested pCG185-1G showed the expected band of 3570 bp when hybridized with the probe (Figure 2A). *Bgl*II cuts only once in the T-DNA portion of the pCG185-1G plasmid in transgenic plants thus the 1051 bp probe would be expected to give hybridization bands of at least 3570 bp depending on the location of other *Bgl*II sites randomly situated in the flanking wheat genomic DNA. The number of bands indicated approximately the number of transgene copies integrated. As indicated in Figures 2B–D, the copy number of the *gusA* gene in each combination were ranged from two to five (1G7B), one to five (5G7B), and two to six (5TGTB154). Transgenic lines transformed with 1G7B contained, on average, 2.6 copies of the *gusA* with three lines containing two copies, two lines contained five copies and one line containing three copies. Transgenic lines transformed with 5G7B contained, on average, 3.3 copies of the *gusA* with one line containing one copy, three lines containing two copies, two lines containing three copies and two lines containing four copies. For 5TGTB154, transgenic lines contained on average, 3.3 copies of the *gusA* with five lines containing three copies, and other three each containing three, four and five copies respectively. There were no significant differences in average transgene copy number between these three combinations: 1G7B (2.6), 5G7B (3.3), and 5TBTG154 (3.3) (ANOVA, *F* = 0.940, *P* = 0.408; Figure 2E). This indicated that the additional *vir* gene(s) did not affect the level of T-DNA integration or copy number into the plant genome as observed by Vain et al. (2004).

### Presence of vector backbone sequences in T_0_ transgenic plants derived from different dual binary vectors

The independent lines co-expressing both *gusA* and *bar* from each vector were tested for the presence of backbone sequences. The presence of plasmid backbone DNA sequence in transgene loci were detected by using three primer sets located outside the left and right T-DNA borders and within the plasmid selectable marker gene (*NPTI*) as indicated in Figure 1. The results of backbone integration in the transgenic lines derived from each vector were summarized in Table 2. For 1G7B, one of six lines contained sequence both flanking the LBs and reaching to the *NPTI* gene, whereas no backbone sequence outside RB border was detected. For 5G7B, three of 24 lines contained sequences both flanking the LBs and reaching to the *NPTI* gene, with two lines having entire backbone integrated. For 5BTG154 with the structure of RB-*gusA*-LB-*bar*, there were eight lines that yielded the PCR fragment flanking LBs among 13 lines investigated, with eight lines having *NPTI* region and six lines containing the entire backbone. For 5LBTG154, there were 10 lines that yielded the PCR fragment flanking left borders, with eight lines covering *NPTI* region and six lines containing the entire backbone. For 5TBTG154, there were six lines that yielded the PCR fragment flanking LBs, with four lines covering *NPTI* region and three lines having the entire backbone. Compared to the backbone integration frequencies of 50.0% for 5LBTG154 which contains single LB in T-DNA, the presence of double LBs minimized the insertion of superfluous DNA sequences into plant genome with observed backbone integration frequencies of 16.7, 12.5, and 20.0% for 1G7B, 5G7B, and 5TBTG154 vectors, respectively. Furthermore, the frequencies of transgenic lines containing backbone sequences flanking the RB were 0, 8.3, 46.2, 30.0, and 10.0% for 1G7B, 5G7B, 5BTG154, 5LBTG154, and 5TBTG154, respectively, lower than the integration of backbone sequences flanking the LB (Table 2). This indicated that the integration of vector backbone DNA sequences is indeed more frequently linked to the LB than to the RB. Moreover, the integration frequencies of bacterial sequences (*NPTI*) in the lines derived from these vectors were 16.7, 12.5, 61.5, 40.0, and 13.3%, respectively.

**Table 2:** **The presence of vector backbone sequence in transgenic lines derived from different vector combinations**.

**Vector combination**	**No. of T_0_ plants co-expressing both *gusA* and *bar***	**Percentage of the presence of backbone sequence**		
		**LB**	***NPTI***	**RB**
1G7B	6	16.7% (1/6)	16.7% (1/6)	0 (0/6)
5G7B	24	12.5% (3/24)	12.5% (3/24)	8.3% (2/24)
5BTG154	13	61.5% (8/13)	61.5% (8/13)	46.2% (6/13)
5LBTG154	20	50.0% (10/20)	40.0% (8/20)	30.0% (6/20)
5TBTG154	30	20% (6/30)	13.3% (4/30)	10.0% (3/30)

### Transgene segregation in T_1_ progenies

The selected T_0_ independent transgenic lines were grown to maturity and self-pollinated seeds were obtained. All of these transgenic lines were morphologically normal and fully fertile. To study inheritance patterns of transgene loci, T_0_ plants co-expressing both *gusA* and *bar*, these were 6, 24, 13, 20, and 34 T_0_ plants derived from different vector combinations (Table S1), were selected for segregation analysis in T_1_ progenies by GUS assay. In transgenic lines which the T_1_ segregation ratio of *gusA* did not match, PCR on genomic DNA was used to check the presence of *gusA*. The results of *gusA* segregation in T_1_ progenies derived from different vector combinations were as summarized in line f, Table 1. For more details, please check Table S1. The trend of segregation ratio was almost the same for all five vectors which was 27–40% with 3:1 segregation, 25–50% with 15:1 segregation, 17–23% with 63:1 segregation, 0–6% for all others. For other segregation types, the lines showed a non-Mendelian segregation ratio of 1:1 or 1:2, presumably due to aberrant gamete or seed formation.

### Recovery of marker-free transgenic wheat plants in T_1_ progenies

**Figure 3:**
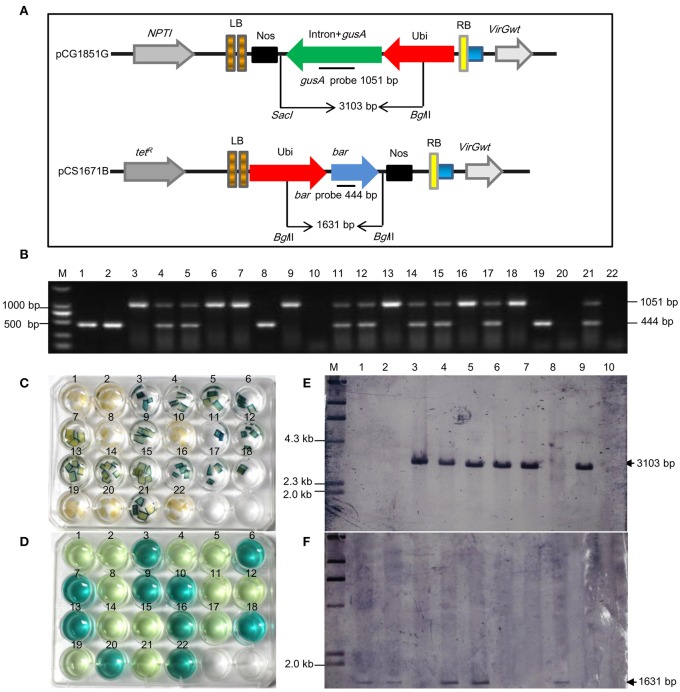
**Segregation of *gusA* and *bar* transgenes in T_1_ progenies of E02-2-4 T_0_ independent line derived from 5G7B vector combination. (A)** Schematic show of the *gusA* and *bar* gene expression cassettes, the location of *Bgl*II and *Sac*I restriction sites, and T-DNA borders. **(B)** PCR detection of *gusA* and *bar* transgenes in T_1_ progenies of E02-2-4 T_0_ plant. **(C)** GUS expression assay. **(D)** PAT assay. The presence of BAR is able to assimilate ammonium when treated with phosphinothricin acetyltransferase (PAT), led to the absence of ammonium ions in medium, thus yield a pale yellow reaction. In contrast, non-transgenic plants without the *bar* gene are not able to assimilate ammonium and result in a jewelry blue or dark blue color. **(E)** Southern blot analysis of *gusA* gene. **(F)** Southern blot analysis of *bar* gene. M, λ-*Hind*III ladder. Lanes 1–10, T_1_ progenies. Genomic DNA and plasmid were digested with both *Sac*I and *Bgl*II, respectively, which produced 3103 bp *gusA* fragment and 1631 bp *bar* fragment, hybridized with a 1051 bp *gusA* probe and 444 bp *bar* probe, respectively. The data presented in **(B–D)** was obtained by analyzing the same set of 21 plants. Line 22, non-transgenic control.

Two transgenes can be separated from each other in subsequent generation of the co-transformants through genetic segregation when they integrated at unlinked genomic loci. The segregation of *gusA* and *bar* were analyzed by PCR, GUS, and PAT protein assays in T_1_ progenies derived from self-pollination of independent T_0_ transgenic plants. The results were as indicated in Table 1. The frequencies of segregation of *gusA* and *bar* in the T_1_ progenies of co-expressing T_0_ independent lines derived from 1G7B, 5G7B, 5BTG154, 5LBTG154, and 5TBTG154 vectors were 16.6, 29.2, 38.4, 45.0, and 30.0%, respectively. Detailed segregation ratios of *bar* and *gusA* in T_1_ progenies of these above T_0_ plants derived from each vector were as listed in Table S1. The frequencies of marker-free plants obtained in T_1_ progenies derived from five vectors were 13.3, 23.4, 21.4, 25.4, and 19.7%, respectively (Tables 1, 3). As indicated in Table 3, among 188 T_1_ plants from seven T_0_ plants derived from 5G7B combination showing segregation, 23.4% (44/188) were positive for *gusA* and lost the *bar* and thus were selection marker-free (SMF) plants. For one T_0_ line of 1G7B, 13.3% were SMF plants. For 5TBTG154, among the nine T_0_ plants tested, 19.7% of T_1_ progenies were SMF plants. The percentage of SMF plants in the T_1_ progenies derived from 5BTG154 and 5LBTG154 combination were 21.4 and 25.4%, respectively. The segregation of the *bar* and *gusA* transgenes was further confirmed by Southern blot analysis of T_1_ plants. As demonstrated in Figure 3, among 21 T_1_ progenies from E02-2-4 T_0_ plant of 5G7B, four had only *bar* inserted, seven were marker-free plants with *gusA* only, eight had both *bar* and *gusA*, indicating no independent segregation of these loci occurred in formation of T_1_ progenies, and two plants were negative for both *gusA* and *bar* (Figure 3B). Further GUS and PAT activity assay confirmed the PCR results (Figures 3C,D).

**Table 3:** **Frequency of marker-free plants obtained in T_1_ progenies derived from five vector combinations**.

**Vector combinations**	**T_0_ line**	Backbone-free lines^*^	**No. of T_1_ plants tested**	No. of T_1_ plants possessing the *gusA* and/or *bar* genes^**^				**Frequency of marker-free plants (%)**
				***gusA*+ *bar*+**	***gusA*+ *bar*−**	***gusA*− *bar*+**	***gusA*− *bar*−**	
1G7B	X03-1-7	Y	15	9	2	3	1	
Total			15		2			13.3
5G7B	E01-1-2	Y	10	6	2	2	0	
	E02-2-4	Y	21	8	7	4	2	
	E02-4-14	Y	19	11	3	4	1	
	E03-1-11	Y	28	15	4	7	2	
	E03-2-7	Y	30	18	4	2	1	
	E04-1-9	N	42	32	9	1	0	
	E04-1-13	Y	39	23	15	0	1	
Total			188		44			23.4
5BTG154	G02-3-4	N	17	13	2	1	1	
	G02-4-8	Y	27	21	4	0	2	
	G02-4-12	N	14	9	5	0	0	
	G03-1-2	N	26	14	9	1	2	
	G03-4-7	N	28	20	4	0	4	
Total			112		24			21.4
5LBTG154	H01-2-6	Y	20	16	1	1	2	
	H02-1-4	Y	11	5	5	0	1	
	H02-2-8	N	13	8	2	2	1	
	H02-2-4	N	21	7	11	0	3	
	H02-3-10	Y	22	16	4	1	1	
	H02-4-2	Y	27	20	5	2	0	
	H02-4-6	N	29	10	16	0	3	
	H03-1-7	N	37	31	2	1	3	
	H03-2-7	N	5	3	1	0	1	
Total			185		47			25.4
5TBTG154	F01-2-5	Y	13	6	3	3	1	
	F01-2-10	Y	29	16	7	4	2	
	F01-2-12	Y	26	16	3	4	3	
	F01-4-2	N	20	13	2	3	2	
	F02-1-9	Y	26	16	5	2	3	
	F02-4-12	Y	15	12	2	1	0	
	F03-2-5	Y	25	14	9	1	1	
	F04-1-3	N	12	9	1	0	2	
	F04-4-3	N	27	19	6	2	0	
Total			193		38			19.7

* *Y, backbone-free; N, contains backbone*.

** *gus(+)bar(+), presence of both gusA and bar; gus(+)bar(−), presence of only gusA; gus(−)bar(+), presence of only bar; gus(−)bar(−), absence of both gusA and bar*.

The presence of the *gusA* and/or *bar* gene in T_1_ plants were further tested by Southern blot analysis. Total genomic DNA was digested with both *Sac*I and *Bgl*II. *Sac*I and *Bgl*II cut once in the T-DNA portion of both the pCG185-1G plasmid and transgenic plants, respectively, and expected to release an approximate 3103 bp band when hybridizing with the *gusA* probe, whereas *Bgl*II cuts twice in the T-DNA portion of both the plasmid pCS167-1B and transgenic plants, and expected to release an approximate 1631 bp band when hybridized with the *bar* probe (Figure 3A). The blot was first hybridized with *gusA* probe, then stripped, and rehybridized with a *bar* probe (Figures 3E,F). Lack of hybridization with the *bar* probe confirmed the absence of the *bar* gene in these lines. The lack of segregation of the *gusA* and *bar* junction fragments in Southern blot agreed well with the segregation result obtained by PCR analysis.

## Discussion

Along with the increased commercialization and planting of a range of GM crops globally, the biosafety of GM crops has become a hot topic and major public concern. The exclusion of nonessential sequences from transformation vectors and generation of marker-free plants are desirable to improve the biosafety and public acceptance of GM crops. In this study, a clean transgene technology was developed and demonstrated to be feasible in wheat for the first time. This can be achieved either with the two T-DNAs separated in two respective plasmids (1G7B and 5G7B) or two T-DNAs separately located in one dual binary vector (5TBTG154). In addition, we found that by repositioning the SMG in the backbone and leaving only the GOI in the T-DNA region (5BTG154) or repositioning the SMG between one LB and double LBs and leaving only the GOI in the T-DNA region were also able to generate marker-free transgenic wheat plants (5LBTG154) (Figure 1). Evaluation of final transformation efficiencies, co-transformation efficiencies, the frequencies of marker-free transgenic plants, and the integration of backbone sequences in the transgenic lines derived from these vector combinations indicated that two vector combinations (5G7B and 5TGTB154) were more efficient in generation of marker-free transgenic wheat plants with no or minimal integration of backbone sequences in wheat genome (Tables 1, 2).

### Effect of *Vir* gene(s) on transformation efficiency

The infection process of *Agrobacterium tumefaciens* involves a set of chromosome-encoded genes (*chv*) involved in attachment of bacteria to plant cells and Ti plasmid-encoded *vir* genes that function intrans, helping in the generation, transfer and integration of T strands into the plant genome (Hooykaas and Beijersbergen, 1994). *Vir* gene copies or combinations have been introduced to *Agrobacterium* by using alternative Ti plasmids, helper plasmids or additional virulence genes on binary Ti vectors (Vain et al., 2004). Different *vir* gene(s) have been shown to increase tumor formation (Jin et al., 1987), transient gene expression (Liu et al., 1992; Hansen et al., 1994; Ke et al., 2001) as well as stable cell and tissue transformation in different plant species (Komari, 1990; Wenck et al., 1999; Tang, 2003; Vain et al., 2004; Wu et al., 2006). In this study, we took advantages of *vir* gene(s) to improve transformation efficiency. Higher GUS transient expression was observed in the combinations of 5G7B which contains an additional *virGwt* gene in GOI plasmid, and 5BTG154, 5LBTG154, and 5TBTG154 which contain *virGwt* in pCLEAN-G based vectors and *virB/C/G* in help plasmid PAL154 (Figure 1). Except for 5BTG154 and 5LBTG154 combinations which contained a structure of “RB-*gusA*-2LB-*bar*” in pCG185-2G plasmid and “RB-*gusA*-2LB-*bar*-LB” in pCG185-3G plasmid, respectively, and matched with a helper plasmid PAL154 (Figure 1), in which the counter-screening of backbone and generation of RB-*gusA*-2LB structure might lost after two rounds of selection, 5G7B and 5TBTG154 combinations had a relatively higher final transformation efficiencies compared with other three vector combinations (Table 1). The addition of *virGwt* gene in the backbone sequence nearly doubled the transformation efficiency when 1G7B (1.5 ± 0.3%) and 5G7B (2.8 ± 0.8%) were compared as observed by Vain et al. (2004) (Table 1), and additional Komari fragment in the help plasmid in 5TBTG154 improved the transformation efficiency to reach at as higher as 4.0 ± 2.1% (Table 1). However, as indicated in Figure 2E, additional *vir* gene(s) did not affect the level of T-DNA integration into the genome of transformed plants as observed in transgenic tobacco and rice plants (Srivatanakul et al., 2000; Vain et al., 2004).

### *Agrobacterium*-mediated T-DNAs co-transformation to generate marker- and backbone-free transgenic wheat plants

Co-transformation of a gene of SMG and a GOI and subsequent segregation of the GOI from the unlinked SMG in the T_1_ generation is a simple and effective alternative strategy for removal of SMG from transgenic plants (Schocher et al., 1986). *Agrobacterium*-mediated T-DNAs co-transformation and segregation of transgenes is potentially a simple process, except that the efficiency of co-transformation and unlinked T-DNAs integration must be high. The efficiency of co-transformation was significantly higher with vectors with two (or more) T-DNA regions, in which each T-DNA is flanked by a left and a right border, in one single *Agrobacterium* strain (one-vector / one-strain) compared with co-inoculation of plant cells with two separate *Agrobacterium* strains, each containing a simple binary vector with a single T-DNA flanked by a right and a left border (two-vector/two-strain) (Komari et al., 1996; Miller et al., 2002). In addition, genetic analysis showed that the T-DNAs derived from different *Agrobacterium* strains were frequently integrated at the same locus, independent of the plant species or transformation method used (De Neve et al., 1997). Therefore, we chose the two T-DNAs in one-vector/one-strain (5TGTB154) and two-vector/one-strain (1G7B and 5G7B) strategies to perform *Agrobacterium*-mediated co-transformation of wheat. Co-transformation efficiencies of 1G7B, 5G7B, and 5TGTB154 were 37.5, 56.3, and 63.0%, and marker-free transgenic progenies were obtained at a frequency of 13.3, 23.4, and 19.7%, respectively (Table 1). Therefore, although the frequencies of co-transformation and marker-free progeny were much lower than that of maize which were 86.7 and 64.4%, respectively, by using one-vector/one-strain method (Miller et al., 2002), comparable frequencies of co-transformation and generation of marker-free progeny in T_1_ generation were obtained compared with a two-vector-/one-strain approach in rice with the 71% (131/185) co-transformation frequency and 44% (4/9) T_0_ lines containing unlinked T-DNA integration (Afolabi et al., 2004) and with 73.6% co-transformation and progeny of three of five T_0_ plants generated marker-free plants in barley (Holme et al., 2012) and a one-vector/one-strain approach in barley with a co-transformation frequency of 66%, of which 24% (15/63) containing segregated GOI inserts (Matthews et al., 2001) and maize with a co-transformation frequency of 67.3% (177/263), of which 16.0% (28/175) containing unlinked events (Huang et al., 2004). The long transfer events in one-vector / one-strain approach might occur due to skipping of the left border and consequent linked co-delivery of T-DNAs of GOI and SMG. As indicated in this study, although the co-transformation efficiency of 5TGTB154 was higher than that of 1G7B and 5G7B due to the two T-DNAs in one vector and matched with a help plasmid with *virB/C/G*, the frequency of marker-free progeny was lower than that of 5G7B. It was reported that placing the GOI on a shorter T-DNA and the SMG on a longer T-DNA increased co-transformation efficiency up to 100% (McCormac et al., 2001). Therefore, it is worthy to try this strategy in the future.

Except for the SMGs, the presence of backbone sequences and/or bacterial DNA sequences in transgenic plants would also be a major biosafety concern. Although right borders can terminate T-strands (Lu et al., 2001) and left borders can initiate T-strands (Horsch and Klee, 1986; Jen and Chilton, 1986; Joersbo and Okkels, 1996), right borders seem to enhance initiation, while left borders appear to attenuate initiation and/or enhance termination (Wang et al., 1987). For most applications, T-strand termination at the left border is desired to prevent vector backbone sequences from being transferred to the plant cell. However, the transfer of backbone sequences could be due to the failure to terminate the T-strand at the left border (read through), or due to the initiation of a T-strand at the left border. In this study, the integration of backbone sequences as detected by PCR for the presence of *NPTI* were 16.7, 12.5, and 13.3% for 1G7B, 5G7B, and 5TBTG154, respectively (Table 2), whereas 30% of events contained backbone sequences in transgenic maize generated by using an ordinary T-DNA with single RB and LB (Huang et al., 2004), indicating double LBs reduced backbone transfer as observed in rice and *Arabidopsis* (Kuraya et al., 2004; Podevin et al., 2006). In addition, although double LBs were used in this study, the frequencies of integration of backbone sequences near the LB were obviously higher than these of near the RB (Table 2). The presence of consensus RB (25 nt) and the overdrive sequence (24 nt) directly outside the RB in these pCLEAN based dual binary vectors might enhance the initiation of a T-DNA strand at the RB and terminate at the LB as the integration of backbone sequences near LB was more frequently than that of RB (Peralta et al., 1986; Van Haaren et al., 1987; Toro et al., 1988; Veluthambi et al., 1988; Hansen et al., 1992; Thole et al., 2007).

### Taking advantages of backbone transfer to generate marker-free transgenic lines

In a widely accepted model, T-strand, the intermediate of T-DNA, is initiated at the RB and terminated at the LB and transferred to plants (Huang et al., 2004). However, a number of reports showed that transfer of backbone segments linked to the T-DNA across the LB to plants are common (Martineau et al., 1994; Ramanathan and Veluthambi, 1995; Van der Graaff et al., 1996; Kononov et al., 1997; De Buck et al., 2000; Yin and Wang, 2000; Sallaud et al., 2003; Kuraya et al., 2004). The read-through as well as initiation at the LB can be responsible for transfer of vector backbone sequences to the plant genome. A simple binary vector with a single right border and a single left border, with the plant selectable marker gene located in the backbone region, can be used to efficiently obtain marker-free plants (Huang et al., 2004). We here also took advantage of the occasional transfer of backbone sequences that occurs during *Agrobacterium*-mediated transformation to efficiently generate marker-free transgenic wheat plants. In two vector combinations, 5BTG154 and 5LBTG154, SMG gene *bar* was located either on the backbone or between the double LBs and an additional LB, whereas the *gusA* cassette was placed between RB and the double LBs (Figure 1). For 5BTG154, SMG located outside the T-DNA (in the vector backbone) to be co-transformed with GOI located inside the T-DNA. When T-DNA and T-DNA plus backbone fragments integrate at different loci, progeny plants free of selectable marker gene can be recovered (Huang et al., 2004). For 5LBTG154, a structure of RB-GOI-LB1 or RB-GOI-LB-SMG-LB2 might form during T-DNAs transfer. These two distinct inserts may be independently transferred and integrated into the plant genome, starting either from RB to LB1, or RB to LB2. Once integrated into the plant genome at unlinked locations, progeny plants free of selectable marker gene can be recovered. The co-transformation efficiencies for 5BTG154 and 5LBTG154 were 92.9 and 87.5%, and frequencies of marker-free plants in T_1_ progeny were 21.4 and 25.4%, respectively (Table 1). These two parameters were obviously higher than these of 1G7B, 5G7B, and 5TGTB154. Repositioning the SMG in the backbone and leaving only the GOI in the T-DNA region, a regular two-border binary vector was able to generate marker-free transgenic maize plants more efficiently (93.0 of 35.6% unlinked events) than a conventional single binary vector with two T-DNAs in a single *Agrobacterium* strain (72.0 of 16.0% unlinked events) (Huang et al., 2004). Furthermore, although the additional *virB/C/G* genes were also presented in 5BTG154 and 5LBTG154, the transformation efficiencies were 0.7 ± 0.2 and 1.3 ± 0.2%, respectively, much lower than 4.0 ± 2.1% for 5TBTG154 (Table 1). The relative lower transformation efficiencies and lower frequencies of marker-free progenies observed for 5BTG154 and 5LBTG154 in this study may due to the fact that unlinked insertion of GOI and SMG might cause the loss of events only harboring the GOI during two-round of strict selection in tissue culture. In addition, the presence of double LBs in the T-DNAs of 5BTG154 and 5LBTG154 might reduce the read-through of LBs and thus decreased the transfer of backbone where *bar* was located (Figure 1). Moreover, the integration of backbone sequences as detected by PCR for the presence of *NPTI* indicated that the frequencies of the presence of backbone sequences were 61.5 and 40.0% for 5BTG154 and 5LBTG154, respectively, much higher than 16.7, 12.5, and 13.3% for 1G7B, 5G7B, and 5TBTG154, respectively (Table 2). Therefore, combined with the results of transformation efficiency, co-transformation efficiency, segregation of maker-free progeny and integration of backbone sequences, we would like to propose that two vector combinations (5G7B and 5TGTB154) are more efficient in generation of marker-free transgenic wheat plants with no and/or minimized integration of backbone sequences in wheat genome.

### Exploring alternative strategies to generate marker- and backbone-free GM wheat

SMG- and backbone-free GM wheat can also be achieved through biolistic-mediated co-transformation of minimal-gene-cassettes of target gene and SMG gene (Gadaleta et al., 2008). However, compared with bombardment, there is a general perception that *Agrobacterium*-mediated transformation of wheat is a better option, particularly when regulatory approval for field trials is required, because it has several advantages including the ability to transfer large segments of DNA with minimal rearrangement, lower transgene copy number with few events of gene silencing and the predisposition to integrate into transcriptional active regions of the genome and improved stability of expression over generations (Hiei et al., 1997; Hansen and Wright, 1999; Dai et al., 2001; Wu et al., 2003; Jones et al., 2005; He et al., 2010; Xia et al., 2012). Besides, genome editing technologies offer a great promise for harnessing plant genes and site-specific integration of foreign genes in crop improvement (Li et al., 2012; Shan et al., 2013; Puchta and Fauser, 2014; Sun et al., 2016). Genome editing can enable efficient and precise genetic modifications by inducing targeted DNA double-strand breaks (DSBs) that stimulate the cellular DNA repair mechanisms, including error-prone non-homologous end joining (NHEJ) to generate mutations and gene knock-outs and homology-directed repair (HDR) to achieve precise gene modifications or foreign gene insertions when a template with regions of homology to the sequence surrounding the DSB is available. Unwanted SMG and/or transgene sequences can be eliminated to obtain the clean edited plants following segregation or by crossing (and back-crossing) of the mutant plants with their parental lines, given that these sequences are not integrated near the targeted locus closely (Ma et al., 2015). However, even though the generation of breaks in both DNA strands induces recombination at specific genomic loci, NHEJ is by far the most common DSB repair mechanism in most organisms, including higher plants, and the frequency of targeted integration by HDR remains much lower than random integration (Puchta, 2005; Puchta and Fauser, 2014). As a result, reports describing successful site-specific trait gene integration through HDR in crop plants are limited (Li et al., 2015; Svitashev et al., 2015; Sun et al., 2016), and not yet in wheat due to its recalcitrance to tissue culture, and especially, a complex genomic background.

Nevertheless, following the improved *Agrobacterium*-mediated transformation efficiency in wheat, which can reach as high as 40–90% (Ishida et al., 2015), the clean transgene technology demonstrated in this study will benefit the production of clean GM wheat plants, and thus simplify the biosafety evaluation process and facilitate the future commercialization of GM wheat to meet diverse challenges in a global context.

## Key concepts

To facilitate the production of clean GM wheat, five advanced dual vectors developed in this study were evaluated for generation of marker- and/or backbone-free transgenic wheat plants via *Agrobacterium*-mediated transformation.

## Author contributions

LQX and HDJ conceived and planned the project. GPW, XDY, and YWS carried out the experiments. LQX and GPW wrote the manuscript. HDJ revised the manuscript. Most of the experiments were carried out at Institute of Crop Sciences, Chinese Academy of Agricultural Sciences (CAAS), Beijing 100081, China.

### Conflict of interest statement

The authors declare that the research was conducted in the absence of any commercial or financial relationships that could be construed as a potential conflict of interest.
