# Supplementary material for: Generation of Marker- and/or Backbone-Free Transgenic Wheat Plants via Agrobacterium-Mediated Transformation
Source: Front Plant Sci. 2016 Sep 21;7:1324. doi: 10.3389/fpls.2016.01324 (PMC5030305; doi:10.3389/fpls.2016.01324)
Supplement: Supplementary file 1 [file Table1.DOCX]

**Table S1.** **Segregation of transgene (*gusA*) in T_1_ progenies of the independent T_0_** plants **derived from different vector combinations**

| Vector combinations | No. of  T_0_ lines (A) | Expected *gusA* segregation ratio  (positive plants:negative plants) |  | No. of lines (B) | % (B/A) |
| --- | --- | --- | --- | --- | --- |
| 1G7B | 6 | 3:1 |  | 2 | 33.3% |
|  |  | 15:1 |  | 3 | 50.0% |
|  |  | 63:1 |  | 1 | 16.7% |
|  |  | others |  | 0 | 0.0% |
| 5G7B | 24 | 3:1 |  | 7 | 29.2% |
|  |  | 15:1 |  | 11 | 45.8% |
|  |  | 63:1 |  | 5 | 20.8% |
|  |  | others |  | 1 | 4.2% |
| 5BTG154 | 13 | 3:1 |  | 4 | 30.8% |
|  |  | 15:1 |  | 6 | 46.2% |
|  |  | 63:1 |  | 3 | 23.1% |
|  |  | others |  | 0 | 0.0% |
| 5LBTG154 | 20 | 3:1 |  | 8 | 40.0% |
|  |  | 15:1 |  | 7 | 35.0% |
|  |  | 63:1 |  | 4 | 20.0% |
|  |  | others |  | 1 | 5.0% |
| 5TBTG154 | 34 | 3:1 |  | 9 | 26.5% |
|  |  | 15:1 |  | 16 | 47.1% |
|  |  | 63:1 |  | 7 | 20.6% |
|  |  | others |  | 2 | 5.9% |

Note: No. of lines showed expected *gusA* Mendelian segregation ratio was tested by χ^2^ test.
