# Supplementary material for: Generation of Marker- and/or Backbone-Free Transgenic Wheat Plants via Agrobacterium-Mediated Transformation
Source: Front Plant Sci. 2016 Sep 21;7:1324. doi: 10.3389/fpls.2016.01324 (PMC5030305; doi:10.3389/fpls.2016.01324)
Supplement: Supplementary file 2 [file Table2.DOCX]

**Table S2.** **The primer sets used in vector construction**

| Amplified fragment | Primer name | Sequence (5’-3’) | Annealing temperature (^o^C) | Size (bp) |
| --- | --- | --- | --- | --- |
| *bar*-cassette | Fu*bar*(CACC) | caccTCACCCAGTGCAGCGTGAC | 56 | 2940 |
|  | Rt*bar* | TGTACAAGAAAGCTGGGTCGG |  |  |
| Ubi:*bar*:nos | Fu*bar* | gtttaaacTCACCCAGTGCAGCGTGAC | 58 | 2936 |
|  | Rt*bar* | gtttaaacTGTACAAGAAAGCTGGGTCGG |  |  |
| LB-Ubi:*bar*:nos | Flb | gtttaaacGTCGAGATGGATCTTGGCAG | 56 | 3054 |
|  | Fu*bar* | gtttaaacTCACCCAGTGCAGCGTGAC |  |  |
| 2LB-Ubi:*bar*:nos-RB | F2lb | gtttaaacACTAGGTAAGATCTTGGCAGGA | 55 | 3114 |
|  | Rrb | gtttaaacAGATCCAGATCTCAAACAAACA |  |  |
| Ubi:*gusA*:nos | FugusA | caccGTATCGATAAGCTTGCATGCCT | 59 | 4385 |
|  | RtgusA | GGCCGCTCTAGAACTAGTGGAT |  |  |
| *bar* gene | *bar*F | GTCTGCACCATCGTCAACC | 60 | 1031 |
|  | *bar*R | GAAGTCCAGCTGCCAGAAAC |  |  |
| *gusA* gene | *gusA*F | AGTGTACGTATCACCGTTTGTGTGAAC | 62 | 444 |
|  | *gusA*R | ATCGCCGCTTTGGACATACCATCCGTA |  |  |
