# Supplementary material for: Generation of Marker- and/or Backbone-Free Transgenic Wheat Plants via Agrobacterium-Mediated Transformation
Source: Front Plant Sci. 2016 Sep 21;7:1324. doi: 10.3389/fpls.2016.01324 (PMC5030305; doi:10.3389/fpls.2016.01324)
Supplement: Supplementary file 3 [file Table3.DOCX]

**Table S3. Primer pairs for detection of the presence of the backbone sequences including *NPTI* gene**

| Target | Sequence of primer pairs | Annealing temperature (^o^C) | | Fragment size (bp) | | Detection vector | |
| --- | --- | --- | --- | --- | --- | --- | --- |
| LB1-F  LB1-R | 5'-TCGAGCGCCTTTGCCCACAAC-3'  5'-GGATATGTATCACCGCGTCTTTGAT-3 | 58 | 771 | | 1G7B, 5G7B | |  |
| LB2-F  LB1-R | 5'-GTTAGACTCGTCGACGGCGTTTA-3'  5'-GGATATGTATCACCGCGTCTTTGAT-3' | 60 | 1268 | | 5BTG | |  |
| LB1-F  LB2-R | 5'-TCGAGCGCCTTTGCCCACAAC-3'  5'-TTTCTGGCAGCTGGACTTCA-3' | 60 | 511 | | 5LBTG | |  |
| LB1-F  LB2-R | 5'-TCGAGCGCCTTTGCCCACAAC-3'  5'-TTTCTGGCAGCTGGACTTCA-3' | 60 | 561 | | 5TBTG | |  |
| RB1-F  RB1-R | 5-AACTGTAGAGTCCTGTTGTCAAA-3  5-ACCGCGGATGACAAAAATGC-3 | 59 | 490 | | 5G7B, 5G7B  5LBTG, 5TBTG | |  |
| RB1-F  RB2-R | 5-AACTGTAGAGTCCTGTTGTCAAA-3  5-GGACAGGTATCCGGTAAGCG-3 | 58 | 499 | | 1G7B | |  |
| NPTI-F  NPTI-R | 5-CGATTCCGACTCGTCCAACA-3  5-CGATTGTATGGGAAGCCCGA-3 | 62 | 543 | | *NPT1* | |  |
