# Supplementary figures and images for: Generation of Marker- and/or Backbone-Free Transgenic Wheat Plants via Agrobacterium-Mediated Transformation
Source: Front Plant Sci. 2016 Sep 21;7:1324. doi: 10.3389/fpls.2016.01324 (PMC5030305; doi:10.3389/fpls.2016.01324)

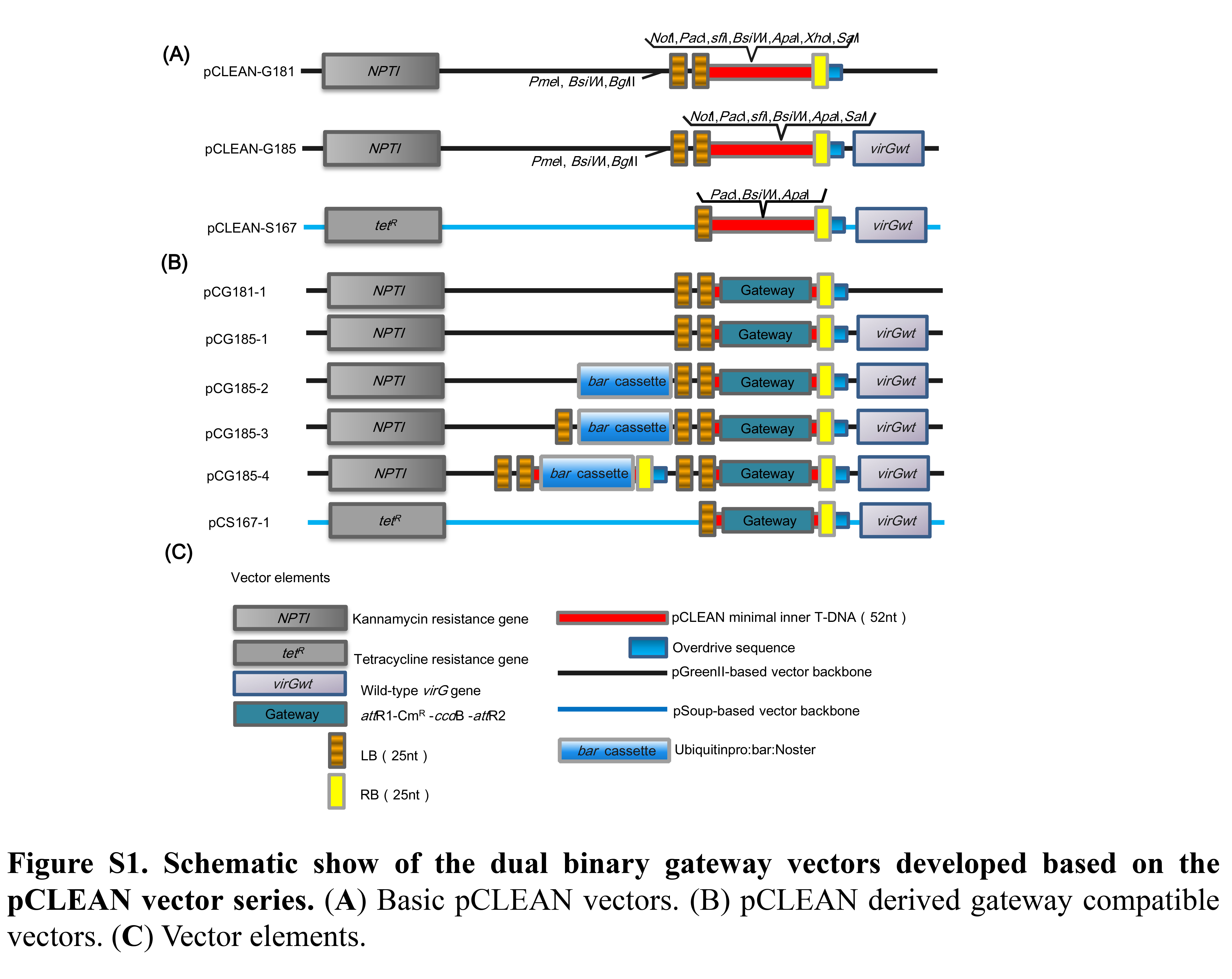

Supplement: Supplementary file 4 [file Image1.JPEG]

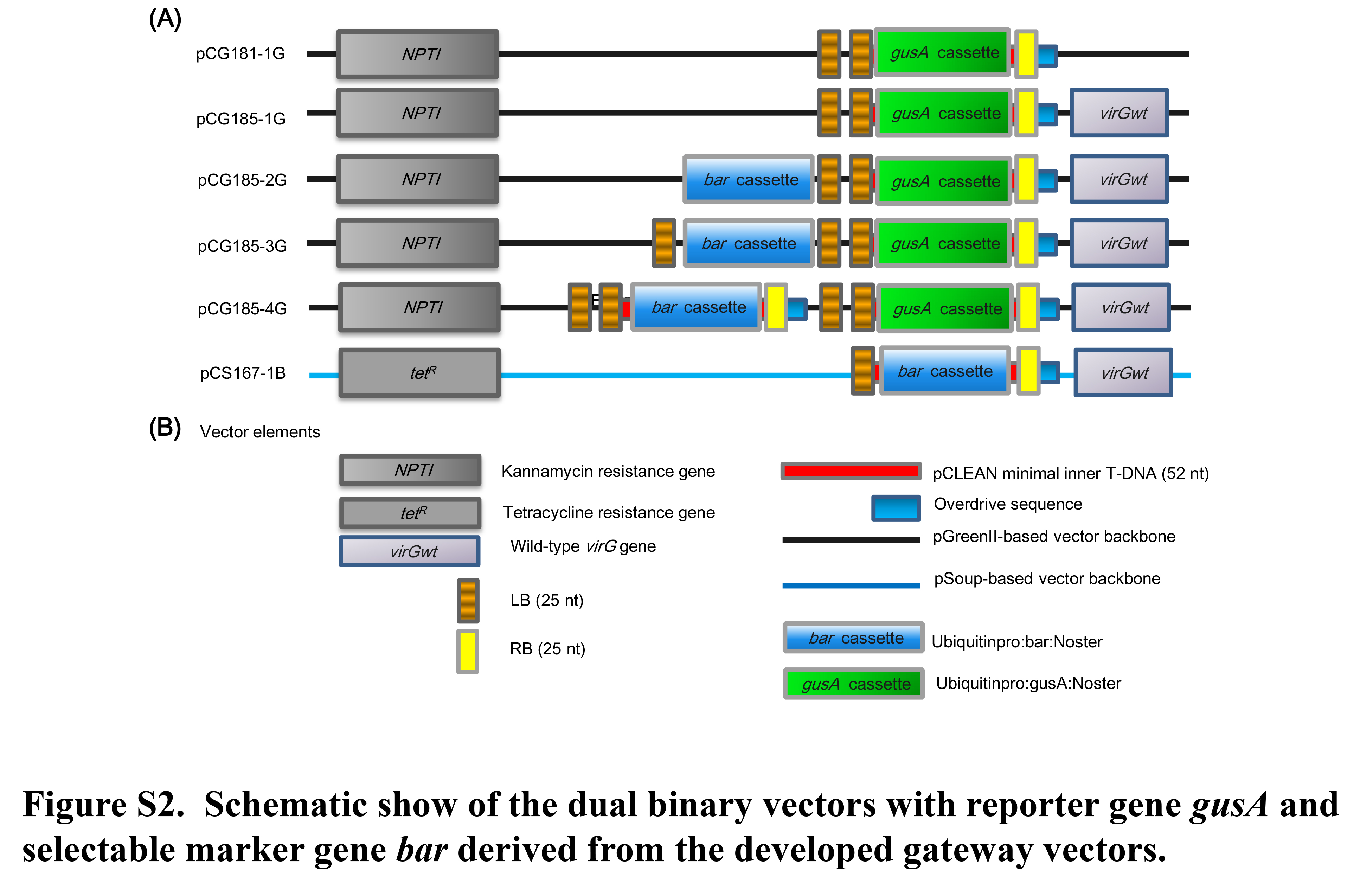

Supplement: Supplementary file 5 [file Image2.JPEG]

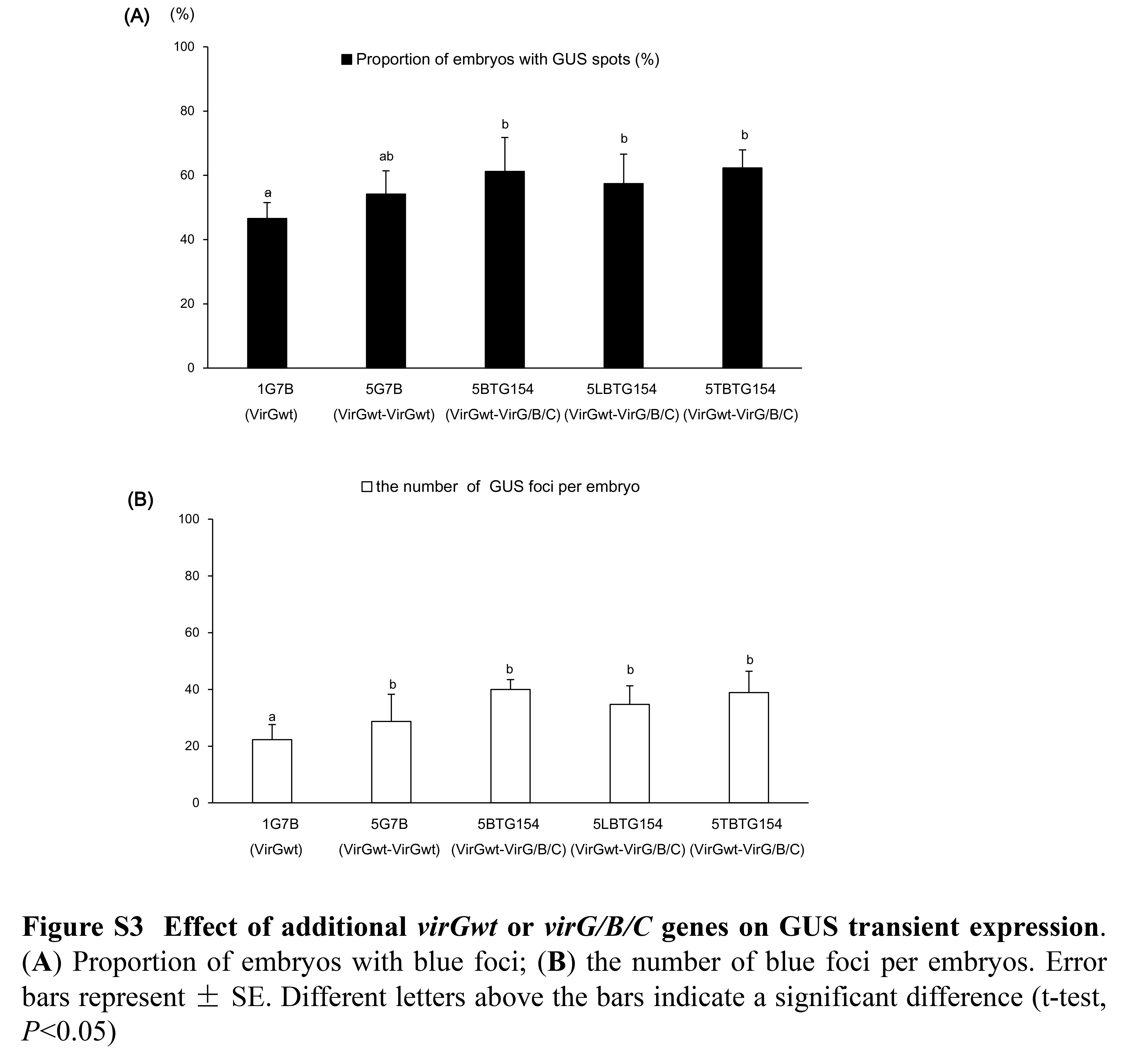

Supplement: Supplementary file 6 [file Image3.JPEG]

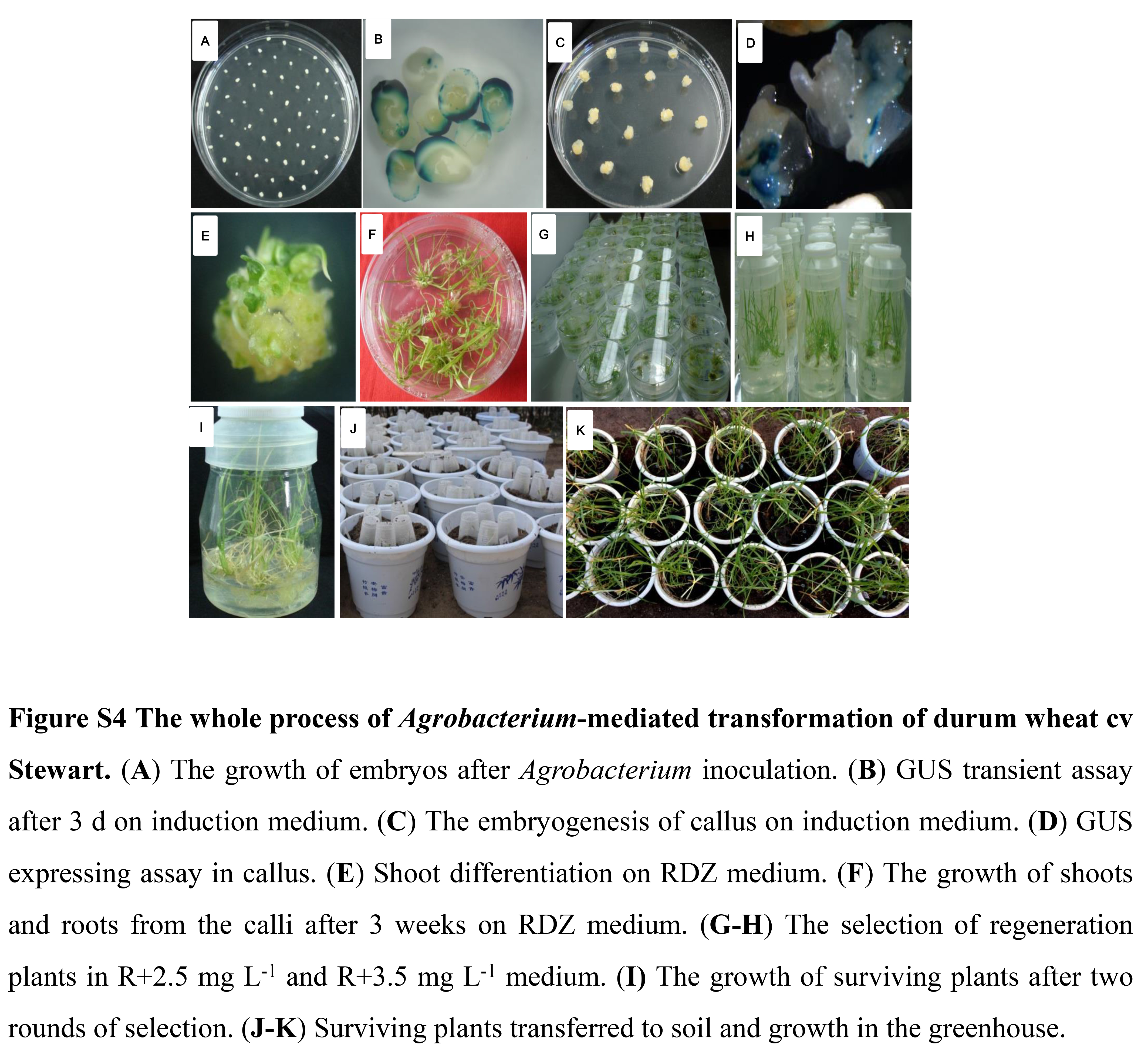

Supplement: Supplementary file 7 [file Image4.JPEG]
